# A WNT protein therapeutic improves the bone-forming capacity of autografts from aged animals

**DOI:** 10.1038/s41598-017-18375-x

**Published:** 2018-01-08

**Authors:** Tao Chen, Jingtao Li, Luis A. Córdova, Bo Liu, Sylvain Mouraret, Qiang Sun, Benjamin Salmon, Jill Helms

**Affiliations:** 1 Stomatological Hospital of Chongqing Medical University, Chongqing Key Laboratory of Oral Diseases and Biomedical Sciences, Chongqing Municipal Key Laboratory of Oral Biomedical Engineering of Higher Education, Chongqing, 400000 China; 2 Division of Plastic and Reconstructive Surgery, Department of Surgery, Stanford School of Medicine, Stanford, CA 94305 USA; 3 State Key Laboratory of Oral Diseases & National Clinical Research Center for Oral Disease & Department of Oral Maxillofacial Surgery, West China Hospital of Stomatology, Sichuan University, Chengdu, 610007 China; 4 Department of Oral and Maxillofacial Surgery, Faculty of Dentistry, University of Chile, Santiago, Chile; 5 Ankasa Regenerative Therapeutics, Inc. 329 Oyster Point Blvd. Suite 3306, South San Francisco, CA 94080 USA; 6 Department of Periodontology, Service of Odontology, Rothschild Hospital, AP-HP, Paris 7 – Denis, Diderot University, U.F.R. of Odontology, Paris, France; 7 Department of Plastic Surgery, The First Hospital of China Medical University, Shenyang, 110001 China; 8 Paris Descartes - Sorbonne Paris Cite University, Dental School, EA2496, Montrouge, France and Dental Medicine Department, Bretonneau Hospital, HUPNVS, AP-HP, Paris, France

## Abstract

Autografts tend to be unreliable in older patients. Some of these age-related skeletal changes appear to be attributable to a decline in endogenous WNT signaling. We used a functional *in vivo* transplantation assay to demonstrate that the bone-forming capacity of an autograft can be traced back to a Wnt-responsive cell population associated with the mineralized bone matrix fraction of a bone graft. Micro-CT imaging, flow cytometry and quantitative analyses demonstrate that this mineralized fraction declines with age, along with a waning in endogenous Wnt signaling; together these factors contribute to the age-related deterioration in autograft efficacy. Using a lipid formulation to stabilize the hydrophobic WNT3A protein, we demonstrate that osteogenic capacity can be restored by incubating the bone graft *ex vivo* with WNT3A. Compared to control bone grafts, WNT-treated bone grafts give rise to three times more bone. These preclinical results establish a pivotal role for WNT signaling in the age-related decline of autologous bone grafting efficacy, and demonstrate a means to restore that efficacy via local, transient amplification of endogenous Wnt signaling.

## Introduction

Autologous bone grafts (autografts) are the standard of care for the majority of musculoskeletal procedures requiring bone regeneration^1^. Autografts are typically collected as part of a surgical procedure where the cortex of bone is harvested along with marrow aspirate, then placed on a back table until needed at the site of reconstruction^2^. Clinicians generally accept that an inverse relationship exists between the quality of the patient’s bone, and the amount of autologous bone graft required to achieve successful healing^3^. In elderly patients and those with metabolic diseases a greater volume of bone graft is thought to be required to achieve bony unions^4^. For example, clinical data indicate that an increase in the patient’s age is accompanied by a decrease in autograft efficacy^5,6^. In experimental animals^7^ an age-related fatty degeneration of bone marrow is accompanied by a decrease in the proportion of osteoprogenitor cells relative to adipoprogenitor cells^8,9^.

Here, we evaluated how aging specifically affects the osteogenic capacity of bone grafts. We developed a functional transplantation assay to pinpoint the component(s) of the graft that gave rise to the new bone. The contribution of stem/progenitor cells to autograft efficacy was also analyzed, and the role that Wnt responsive cells play in the osteogenic capacity of an autograft was explored. Collectively these data provide insights into the molecular and cellular basis for the osteogenic capacity of bone grafts and how autograft efficacy may be restored by an *ex vivo* treatment using a WNT protein therapeutic.

## Methods and materials

### Animals

Stanford APLAC approved all procedures (#13146), which conform to ARRIVE guidelines. *C57BL/6* and *Axin2* ^*LacZ/+*^ mice were obtained from Jackson Laboratory (Bar Harbor, ME). *Axin2* ^*CreERT2/+*^; *R26* ^*mTmG/+*^ mice were generated from *Axin2* ^*CreERT2/+*^ and R26^*mTmG/+*^ mice, both from Jackson Labs (#18867 and #7676). Nude mice (#086) were purchased from Charles River and Beta-actin-enhanced green fluorescent protein (ACTB-eGFP) transgenic mice (#027) were purchased from the Jackson Laboratory (Sacramento, California).

### Surgeries

In total, 59 animals were used in this study (see Supplemental Table 1 for numbers of animals used in each procedure). Two types of surgeries were performed: a bone graft harvesting procedure coupled to a mono-cortical tibial defect, and a bone grafting procedure coupled to a sub-renal capsule (SRC) transplantation assay. For all surgeries, an appropriate level of anesthesia was reached via intraperitoneal (IP) injection of Ketamine/Xylazine, followed by shaving and cleansing of the surgical site.

To mimic an autologous bone graft procedure^7^, syngeneic mice were employed. *Axin2* ^*CreERT2/+*^; *R26* ^*mTmG/+*^ mice served as donors. To activate the recombination event and label Wnt responsive cells with GFP, 4 mg/25 g body weight of tamoxifen was delivered via intra-peritoneal injection every day for 5 days. After the labeling period, animals were sacrificed and bone graft was harvested from the femurs and tibiae, and pooled. Syngeneic *Axin2* ^*CreERT2/−*^; *R26* ^*mTmG/+*^ mice served as hosts (these animals were not treated with tamoxifen), and the grafts were then placed into the sub-renal capsule (see below).

Mono-cortical defect model: following anesthesia, a longitudinal cutaneous incision was made over the proximal medial diaphysis to expose the anterior tibial muscle of skeletally mature mice. The muscle attachment to the anterior edge of the tibia was released with a blade and the lateral surface of the tibia was accessed with the aid of a periosteal elevator. A mono-cortical defect that reached the medullary cavity was produced using a high-speed dental engine (15,000 rpm) and 1.0-mm drill bit (Drill Bit City, Chicago, IL, USA). Care was taken not to injure the medial side of the tibia. The tension-free primary closure was achieved using non-resorbable sutures (ETHICON). No adverse events (e.g., uncontrolled pain, infection, prolonged inflammation) were encountered and tissues were collected at the indicated time points.

Sub-renal capsule transplantation: *Syngeneic Axin2* ^*CreERT2/−*^ *; R26* ^*mTmG/+*^ *mice and nude*; mice served as hosts respectively and the transplantation of Axin2 ^LacZ/+^ or the syngeneic *Axin2* ^*CreERT2/+*^; *R26* ^*mTmG/+*^ from tamoxifen labeled donor bone graft followed an established SRC transplant procedure^10^.

### Bone graft treatments

Bone graft was harvested from either young (defined as being < 3 months old) or aged (defined as being > 12 months old; Supplemental Table 1) mice. To collect the bone marrow component of a bone graft, mice were euthanized, tibiae and femurs were dissected, split lengthwise, and bone marrow was gently scraped out using a micro-curette; the material was promptly placed into a 1.7 mL Eppendorf tube containing 1 mL Dulbecco’s Modified Eagle’s Medium with 10% fetal bovine serum and 1% penicillin/streptomycin (DMEM). In experiments where the bone graft was separated into a cellular marrow fraction and a mineralized bone chip fraction, this separation was achieved using gentle centrifugation (e.g., 14 g for 2 min).

In some cases, the cellular marrow fraction was combined with demineralized bone matrix (DBM; Musculoskeletal Tissue Foundation, New Jersey). In these experiments, the marrow fraction was first dissociated by gentle pipetting followed by centrifugation at 14 g for 2 minutes to separate the marrow cells from any remaining bone chips. Bone chips were discarded, the residual cellular material was transferred to a new Eppendorf tube and centrifuged at 200 g for 5 minutes to create a cellular pellet, then the pellet was combined with DBM and transplanted into the sub-renal capsule of a syngeneic host mouse. Samples were harvested at various time points after transplantation, then prepared for analyses as outlined below.

In some experiments, the cellular marrow fraction and a mineralized bone chip fraction were treated with a liposomal formulation of WNT3A protein (L-WNT3A), or an identical liposomal formulation containing PBS (L-PBS). The individual cellular marrow and mineralized matrix fractions were incubated in 200 μL of DMEM then treated with either L-PBS (1x) or L-WNT3A (0.15 µg/mL active WNT3A). After 1 h incubation at 23 °C, a subset of treated samples was moved to new wells containing 1 mL of fresh media; the remaining samples were maintained in the same wells. In all cases, samples were further incubated at 37 °C, 5% CO_2_ for 23 h then washed with PBS and prepared for quantitative analyses (see below).

### Bone marrow stromal cell isolation

To enrich for bone marrow stem/stromal cell populations, bone graft was placed in DMEM supplemented with 10% FBS^11^; after 24 h, cells were separated into non-adherent and adherent populations, with the latter containing the majority of stem/stromal cells^12^. Stro-1 + /CD73 + /CD105 + cells are considered marrow-derived stem cells^13^.

### DNA quantification and quantitative reverse transcription-polymerase chain reaction (qRT-PCR)

DNA was extracted using the DNeasy Tissue Kit (Qiagen) then quantified using Quant-iT PicoGreen dsDNA Kit (Invitrogen) and a microplate fluorescence reader (Berthold) as described^10^. For qRT-PCR, samples were homogenized in Trizol solution and RNA was isolated with RNeasy plus kit (Qiagen). Reverse transcription was performed with SuperScript III First-Strand Synthesis SuperMix for qRT-PCR Kit (Life Technologies). Primers sequences (5′ to 3′) are as follows: *ß-actin*, [for-GGAATGGGTCAGAAGGACTC], [rev-CATGTCGTCCCAGTTGGTAA]; *Osterix*, [for GGAGACCTTGCTCGTAGAT TTC], [rev- GGGATCTTAGTGACTGCCTAAC]; *Runx2*, [for- TGGCTTGGGTTTC AGGTTAG], [rev- CCTCCCTTCTCAACCTCTAATG]; *Axin2*, [for-TCATTTTCC GAGAACCCACCGC], [rev- GCTCCAGTTTCAGTTTCTCCAGCC].

### Imaging analyses

A micro-computed tomography data-acquisition system (RS150; GE Healthcare, Amersham, UK) at 49 μm voxel size was used and analyses followed published guidelines^14^. The distal tibiae and proximal femurs of young (<3 months old) and aged (>12 months old) were scanned the resulting data was exported into Osirix software version 5.8 (Pixmeo, Bernex, Switzerland) and registered for segmentation in the same orientation. Color mapping to reveal cortical and trabecular thickness was obtained from µCT data and measured in millimeters, at the proximal femur.

### Flow cytometry

*Axin2* ^*CreERT2/+*^ *;R26* ^*mTmG/+*^ mice received a daily IP injection of 100 µL tamoxifen suspended in corn oil/10% ethanol for 5 days. The control group received corn oil injections without tamoxifen. On post-injection day 7, mice (N = 5) were sacrificed, tibiae and femurs were collected, and marrow was collected by gentle scraping and/or flushing (using 2 mM EDTA in 2% FBS). Cells were filtered through a 40-μm nylon mesh, pelleted by centrifugation (200 g at 4 °C), washed twice, re-suspended in flow cytometry staining buffer (2% FBS in PBS) and kept on ice for subsequent flow cytometry analyses. Aliquots of the mineralized fraction were gently crushed by mortar and pestle then digested in 0.25% collagenase (StemCell Technologies, Canada) for 45 min at 37 °C under constant agitation, then filtered through a 40-μm nylon mesh, pelleted by centrifugation (200 g at 4 °C), washed twice, re-suspended in flow cytometry staining buffer, and kept on ice until subjected to flow cytometry. Flow cytometry experiments were performed in triplicate, using a FACS AriaII (BD Biosciences); data were analyzed using FlowJo Software (Tree Star, Inc, Eugene, OR).

### Histology

Aniline blue staining was performed. Slides were de-paraffinized in Citrosolv then hydrated through a graded ethanol series to distilled water. Slides were then stained in a saturated solution of picric acid followed by a 5% Phosphotungstic acid solution and staining in 1% Aniline blue. Slides were then dehydrated and cover-slipped.

### Alkaline phosphatase (ALP) activity

The alkaline phosphatase (ALP) substrate is 5-bromo-4-chloro-3-indolyl phosphate (BCIP; Roche, #113832201). To detect ALP activity, BCIP and nitro blue tetrazolium chloride (NBT; Roche, #11383213001) produce an insoluble NBT diformazan end-product that is blue/purple and can be observed visually. Tissue sections were incubated in NBT and BCIP at 37 °C for 30 min then dehydrated in a series of ethanol and xylene and subsequently cover-slipped with Permount mounting media.

### Tartrate-resistant acid phosphatase (TRAP) activity

Tartrate-resistant acid phosphatase (TRAP) activity was observed using a leukocyte acid phosphatase staining kit (catalog #386A-1KT, Sigma-Aldrich, St. Louis, MO). Following paraffin removal and hydration, tissue sections were incubated with TRAP solution mixture for 20–60 min at 37 °C, which generates highly insoluble dye deposits that can be detected visually. After its development, the slides were dehydrated in a series of ethanol and Citrosolv and subsequently cover-slipped with Permount mounting media.

### Immunohistochemistry

Immunostaining was performed using standard procedures^15^. In brief, tissue sections were de-paraffinized following standard procedures. Endogenous peroxidase activity was quenched by 3% hydrogen peroxide for 5 min, and then washed in PBS. Slides were blocked with 5% goat serum (Vector S-1000) for 1 h at room temperature. The appropriate primary antibody was added and incubated overnight at 4 °C, then washed in PBS. Samples were incubated with appropriate biotinylated secondary antibodies (Vector BA-x) for 30 min, then washed in PBS. Antibodies included rabbit polyclonal anti-GFP (Cell Signaling Technology), Runx2, Ki67, Osterix, Col1  and anti-Stro-1 (Abcam), and anti-CD73 and anti-CD105 (BD Biosciences). An avidin/biotinylated enzyme complex (Kit ABC Peroxidase Standard Vectastain PK-4000) was added and incubated for 30 min and a DAB substrate kit (Kit Vector Peroxidase substrate DAB SK-4100) was used to develop the color reaction.

### TUNEL staining

Terminal deoxynucleotidyl transferase dUTP nick end labeling (TUNEL) assays (Roche) were performed as previously described^10^. Tissue sections were photographed using a Leica digital imaging system.

### Xgal staining

To detect Xgal activity, tissues were fixed with 0.2% glutaraldehyde/PBS for 15 min and then washed 3 times with wash buffer containing 0.005% Nonidet P-40, 0.01% sodium deoxycholate, 2 mM MgCl_2_/PBS. Tissue sections were stained overnight at 37 °C in a staining solution containing 5 mM potassium ferricyanide, 5 mM potassium ferrocyanide, 2 mM MgCl_2_, and 1 mg/ml Xgal. Sections were rinsed 2 times in PBS, dehydrated in a graded ethanol series and cleared in CitriSolv, then mounted with Permount^16,17^.

### Histomorphometric analyses

Histomorphometry was performed in Image J software (1.50 b). At least 4 slides from each sample were used for quantification. For apoptosis detection, the number of terminal deoxynucleotidyl transferase dUTP nick end labeling TUNEL-positive (TUNEL^+ve^) cells were counted, and expressed as a percent of the total DAPI^+ve^ viable cells. Aniline blue staining was used for quantification of new bone formation. The area of new bone was used as numerator and the total graft area was used as denominator.

### Statistical analyses

Results were presented as mean ± standard deviation. Two-tailed Student’s t-test was used to determine significant differences between data sets. P value < 0.05 were considered statistically significant and all statistical analyses were performed with Excel software.

### Ethics

The Stanford committee on Animal Research approved all procedures.

### Data Availability

The datasets generated during and/or analysed during the current study are available from the corresponding author on reasonable request.

## Results

### Cells associated with the mineralized matrix of a bone graft are primarily responsible for its osteogenic properties

As a first step towards determining which component of an autograft contributes most to its osteogenic capacity, we separated bone graft into two fractions: a cellular component that primarily consisting of hematopoietic components and stem/progenitor cells^18^; and a mineralized matrix fraction that primarily contained bone chips and mineralized matrix. This rudimentary separation was achieved by subjecting bone graft to gentle centrifugation, sufficient to cause the bone chips to settle to the bottom of the tube.

**Figure 1:**
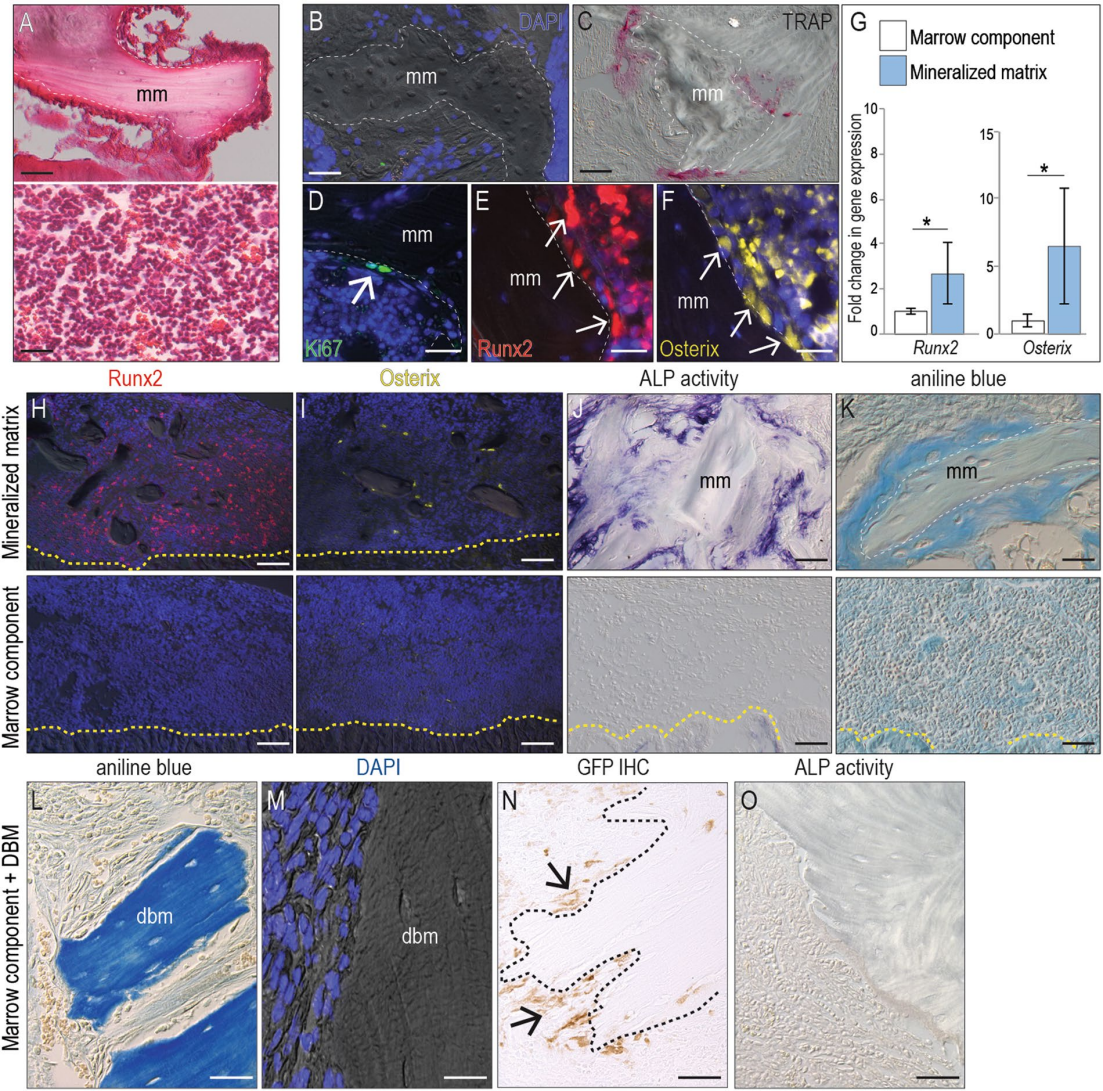
The mineralized component of an autograft is the major contributor to bone regeneration. (**A**) H&E staining of a representative tissue section through the mineralized (top) and marrow (bottom) components of a bone graft. The dotted line demarcates the mineralized matrix (mm). Representative tissue sections through the mineralized component, transplanted to the sub-renal capsule (SRC) and harvested after 5 days then stained (**B**) for DAPI to detect viable cell nuclei, (**C**) TRAP to detect osteoclast activity, (**D**) Ki67 to detect proliferating cells, and (**E**) Runx2 and (**F**) Osterix to detect osteoprogenitor cells. (**G**) Quantification of *Runx2* and *Osterix* expression in the mineralized matrix and marrow components. The osteogenic capacities of the marrow vs. the mineralized component were assessed by transplantation to the SRC. Representative tissue sections immunostained to detect (**H**) Runx2 and (**I**) Osterix expression, and stained to detect (**J**) ALP activity. (**K**) Aniline blue histology identified new bone matrix. Representative tissue sections through demineralized bone matrix (DBM) combined with the marrow component, transplanted to the SRC. After 7 days, tissues were harvested then stained for (**L**) Aniline blue, (**M**) DAPI, (**N**) GFP expression, and (**O**) ALP activity. In all panels the dotted yellow line indicates the boundary between the host kidney and the transplanted material. Abbreviations: as noted in text. Scale bars = 50 µm. Asterisk indicates p-value < 0.05.

Upon collection, histological examination of the two components of the bone graft showed fragments of mineralized matrix that were surrounded by cells that appeared to be attached to the bone, and the marrow component that was primarily comprised of cells and blood (Fig. 1A). The mineralized matrix component was analyzed first: DAPI staining revealed that all the osteocytes in the mineralized matrix were dead (Fig. 1B), even if the time between harvest and transplantation into the sub-renal capsule (SRC) of syngeneic mice was ~15 min. When the mineralized matrix fraction was transplanted to the SRC the necrotic bone was resorbed by TRAP^+ve^ osteoclasts (Fig. 1C).

Cells surrounding the fragments of mineralized matrix, however, were viable (Fig. 1B); we turned our attention to these cells. Immunostaining for the proliferation marker Ki67 suggested that few, if any, of them were mitotically active (Fig. 1D); rather, the cells associated with the mineralized matrix expressed the osteogenic markers Runx2 (Fig. 1E) and Osterix (Fig. 1F). Quantitative RT-PCR analyses were used to verify that the osteogenic genes were expressed at significantly higher levels in the mineralized matrix component compared to the marrow fraction of the bone graft (Fig. 1G).

To directly test the osteogenic potential of each component of the bone graft, the cellular fraction and mineralized matrix fraction were separately transplanted to the SRC. After four days, samples were analyzed for Runx2 and Osterix expression, which revealed that the majority of immunopositive cells were in the mineralized matrix transplants (Fig. 1H,I). After 7 days, only the mineralized matrix transplants showed evidence of alkaline phosphatase (ALP) activity (Fig. 1J) and new bone formation (Fig. 1K). Thus, in comparison to the marrow fraction, the mineralized matrix component of a bone graft exhibited robust osteogenic activity.

### Providing the marrow component of a bone graft with a DBM scaffold does not result in osteogenesis

Was the absence of an osteogenic response from the marrow component because the cells lacked a mineralized scaffold on which they could attach? We tested this possibility by combining the freshly harvested marrow component with demineralized bone matrix (DBM) then transplanting the cells + DBM into the SRC. As before, the mineralized matrix component of the bone graft served as the positive control.

After 7 days, cells + DBM samples were harvested and analyzed. DBM was readily identifiable by aniline blue staining (Fig. 1L). The lack of viable osteocytes within the DBM was confirmed by DAPI staining (Fig. 1M). In this way, DBM resembled the mineralized matrix component of a bone graft in that osteocytes within the mineralized matrix were dead.

Had the marrow cells survived the transplant, and did they remain associated with the DBM? Since the cellular component of the bone graft was harvested from beta actin-GFP donor mice, GFP immunostaining could be used to confirm that viable marrow cells had indeed survived transplantation and remained associated with the DBM (Fig. 1N).

Despite their proximity/attachment to a DBM scaffold, the marrow component still did not give rise to new bone, nor was their evidence of ALP activity (Fig. 1O). Thus, cells that are attached to the mineralized matrix component are primarily responsible for the robust osteogenic potential of a bone graft. Even when provided with a mineralized scaffold, the cellular fraction of a bone graft exhibited minimal osteogenic activity.

### Most Wnt responsive cells in a bone graft associate with the mineralized matrix

**Figure 2:**
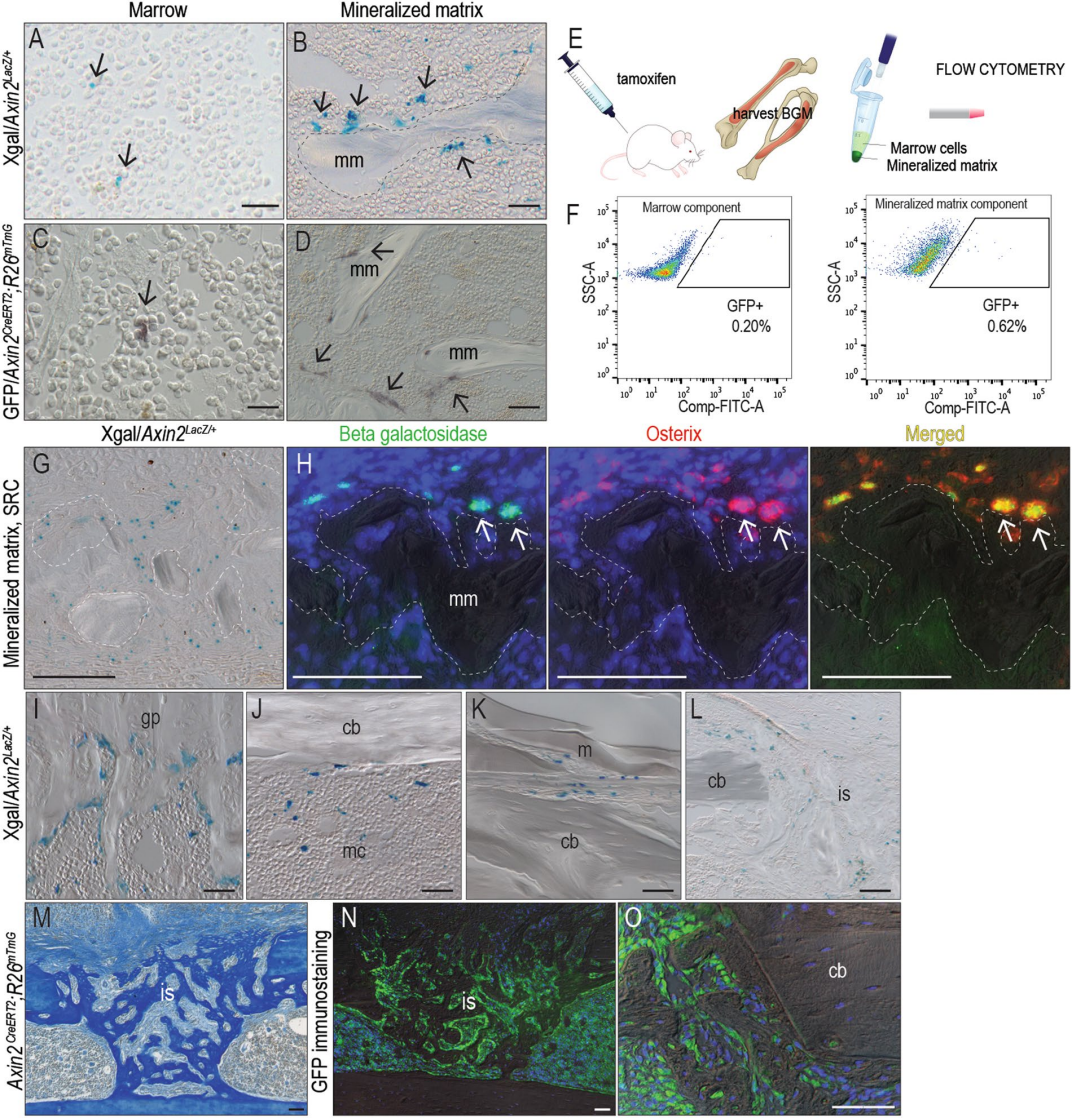
Wnt responsive osteoprogenitor cells associate with the mineralized matrix. (**A**) Representative tissue sections through the marrow component and (**B**) mineralized matrix components of a bone graft, harvested from *Axin2* ^*LacZ/+*^ mice then stained for Xgal to detect Wnt responsive cells. Dotted line indicates the mineralized matrix. (**C**) Representative tissue sections through the marrow and (**D**) mineralized matrix components of a bone graft, harvested from *Axin2* ^*CreERT2/+*^; *R26* ^*mTmG/+*^ mice then immunostained for GFP to detect Wnt responsive cells. (**E**) Experimental design to quantify the distribution of Wnt responsive cells in a bone graft. *Axin2* ^*CreERT2/+*^; *R26* ^*mTmG/+*^ mice received tamoxifen injections for 5 days, after which bone grafts were harvested, separated into marrow and mineralized components, then subjected to flow cytometry to determine the percent of GFP^+ve^ cells in each fraction. (**F**) Of the total cells in the marrow component of the bone graft, 0.2% are GFP^+ve^; of the total cells in the mineralized matrix component, 0.62% are GFP^+ve^. To clarify the identity of the Wnt responsive population, bone grafts were harvested from *Axin2* ^*LacZ/+*^ mice, separated into a mineralized matrix component then transplanted into the SRC. Seven days later (**G**) X-gal staining identified Wnt responsive cells. (**H**) A subset of β-gal^+ve^ Wnt responsive cells co-expressed Osterix. In other locations of the *Axin2* ^*LacZ/+*^ skeleton including (I**) t**he growth plate, (**J**) the endosteum and marrow cavity, (**K**) the periosteum and (**L**) a tibial injury site, Xgal^+ve^ cells localize with sites of new bone formation. Bone injuries elicit robust new bone formation as shown by (**M**) aniline blue histology on post-injury day 7 after the generation of a mono-cortical defect in *Axin2* ^*CreERT2/+*^; *R26* ^*mTmG/+*^ mice. Tamoxifen was delivered on the day of injury (day 0) and (**N**,**O**) GFP immunostaining on post-injury day 7 demonstrating the majority of newly regenerated bone is derived from an initial Wnt responsive population. Abbreviations: cb, cortical bone; en, endosteum; gp, growth plate; m, muscle; mc, marrow cavity; mm, mineralized matrix; is, injury site. Scale bars = 50 µm.

Wnts are pro-osteogenic proteins that regulate human bone mass and fracture healing^19,20^ and our next objective was to determine whether the cells associated with the mineralized matrix were actively responding to an endogenous Wnt signal. Bone graft was harvested from a strain of Wnt reporter mice, *Axin2* ^*LacZ+*^, then separated as into marrow and mineralized matrix fractions. Xgal staining confirmed the presence of Wnt responsive cells in both components, but most Wnt responsive Xgal^+ve^ cells were associated with the mineralized matrix fraction (Fig. 2A,B).

We verified the distribution of Wnt responsive cells in a bone graft using a second lineage-tracing strain of Wnt reporter mice. In *Axin2* ^*CreERT2/+*^; *R26* ^*mTmG/+*^ mice, tamoxifen was given for 5 days then bone graft was harvested and analyzed. As observed previously, most Wnt responsive (GFP^+ve^) cells were associated with the mineralized matrix fraction (Fig. 2C,D).

Fluorescence activated cell sorting was used to quantify the percent of Wnt responsive cells in the marrow and mineralized matrix fractions (Fig. 2E). Bone graft from *Axin2* ^*CreERT2/+*^; *R26* ^*mTmG/+*^ mice was subjected to flow analyses, which demonstrated that most Wnt responsive GFP^+ve^ cells were associated with the mineralized matrix (Fig. 2F). A subset of these Wnt responsive cells expressed osteogenic markers. This was demonstrated by harvesting bone graft from *Axin2* ^*LacZ/+*^ mice then transplanting the material to the SRC. Xgal staining (Fig. 2G) and co-immunostaining for beta galactosidase and Osterix (Fig. 2H) confirmed that some Wnt responsive cells were osteoprogenitors. Wnt responsive osteoprogenitor cells were also identified at sites of new bone formation (Fig. 2I,J,K) and at a site of bone repair (Fig. 2L).

Wnt responsive osteoprogenitor cells directly contributed to bone regeneration. We demonstrated this by producing skeletal injuries in *Axin2* ^*CreERT2/+*^; *R26* ^*mTmG/+*^ mice then injecting tamoxifen on the day of injury. The defect site was assessed for GFP immunostaining on post-surgery day 7, when bone healing is underway. The bony regenerate (Fig. 2M) was largely comprised of GFP^+ve^_,_ Wnt responsive cells (Fig. 2N,O). In most cases the GFP^+ve^ cells were located immediately adjacent to collagen type I^+ve^ bone matrix (Supplemental Fig. 1A–C). Thus, Wnt responsive cells were predominantly found in the mineralized matrix of a bone graft and after injury, the progeny of these osteoprogenitor cells were responsible for bone healing.

### The age-related reduction in autograft efficacy can be traced back to a decline in both bone density and endogenous Wnt signaling

**Figure 3:**
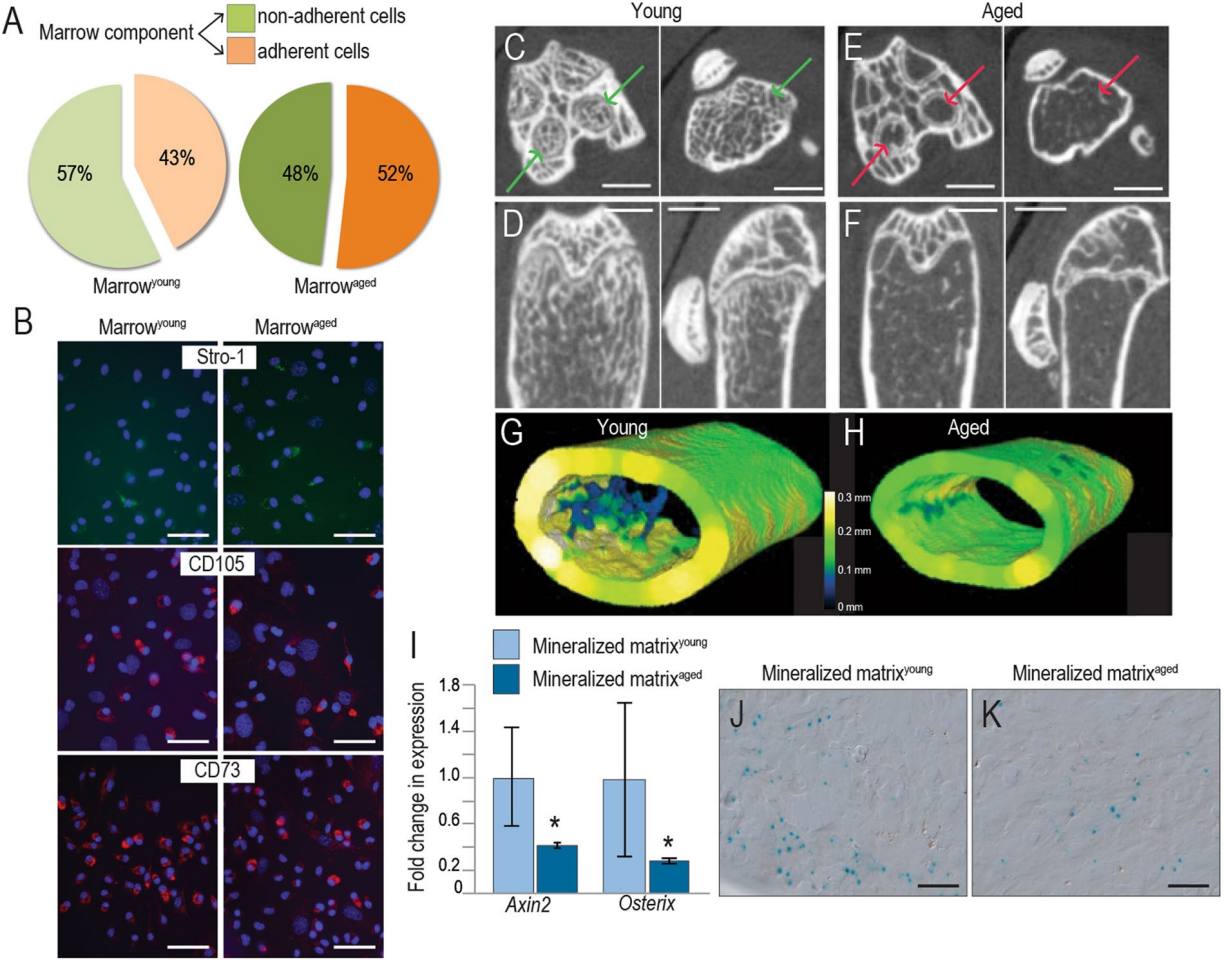
Aging impacts the Wnt responsiveness and osteogenic potential of BGM. (**A**) Bone grafts were harvested from young and aged mice and the marrow component was isolated then cultured to separate non-adherent from the adherent cell population containing stromal/stem cells. The total number of cells in each fraction was calculated^47^. In these cultured cell populations (**B**) immunostaining for Stro-1, CD105, and CD73 was performed. (**C**,**D**) Micro-CT imaging of the skeletons of young and (**E**,**F**) aged mice. Color mapping of cortical and trabecular thicknesses, obtained from µCT data, are shown from representative (**G**) young and (**H**) aged mice. (**I**) Quantitative RT-PCR expression of *Axin2* and *Osterix* in the mineralized matrix components of bone grafts harvested from young (light blue bars) and aged (dark blue bars) mice. Xgal staining of the marrow from (**J**) young and (**K**) aged *Axin2* ^*LacZ/+*^ mice. Scale bars = 50 µm.

Clinicians have long recognized that the osteogenic capacity of a bone graft declines with a patient’s age, to such an extent that autografting in individuals >65 years of age is considered unreliable and ineffective^21^. Some reports suggest that the percentage of marrow-derived stem cells decline with age^22^ but we found that the percentage of stem/stromal cells was not significantly different between young (43%) and aged (52%) animals (Fig. 3A). Immunostaining for the stem/stromal markers Stro-1, CD105, and CD73 also did not reveal any obvious age-dependent differences in expression (Fig. 3B).

The age-related loss in osteogenic capacity of a bone graft has also been associated with a loss in mineralized matrix^23,24^. Cross-sectional and longitudinal micro-CT imaging of young (<3-month-old (Fig. 3C,D) and aged (>12-month-old, Fig. 3E,F) long bones illustrated this loss in trabeculation, which was quantified by volume rendering and color mapping. In young animals (Fig. 3G) the average trabeculae were 0.1–0.2 mm thick whereas in aged mice (Fig. 3H) the trabeculae were so thin that they fell below the limit of detection by micro-CT.

Given that the majority of Wnt responsive cells were associated with the mineralized matrix, and the amount of mineralized matrix declines with age, we hypothesized that the Wnt responsive status of the bone graft would also be reduced in aged animals. Indeed, qRT-PCR analyses demonstrated a significant reduction in expression of the Wnt target gene *Axin2*, coincident with a drop in *Osterix* expression (Fig. 3I). Xgal staining confirmed that in the mineralized matrix the number of Wnt responsive cells was reduced because of age (Fig. 3J,K). Thus, the age-related decline in autograft efficacy correlates with a deterioration in endogenous Wnt signaling, and with a loss in trabecular bone material that contributes to the mineralized fraction of an autograft.

### A WNT therapeutic recharges autologous bone grafts from aged animals

**Figure 4:**
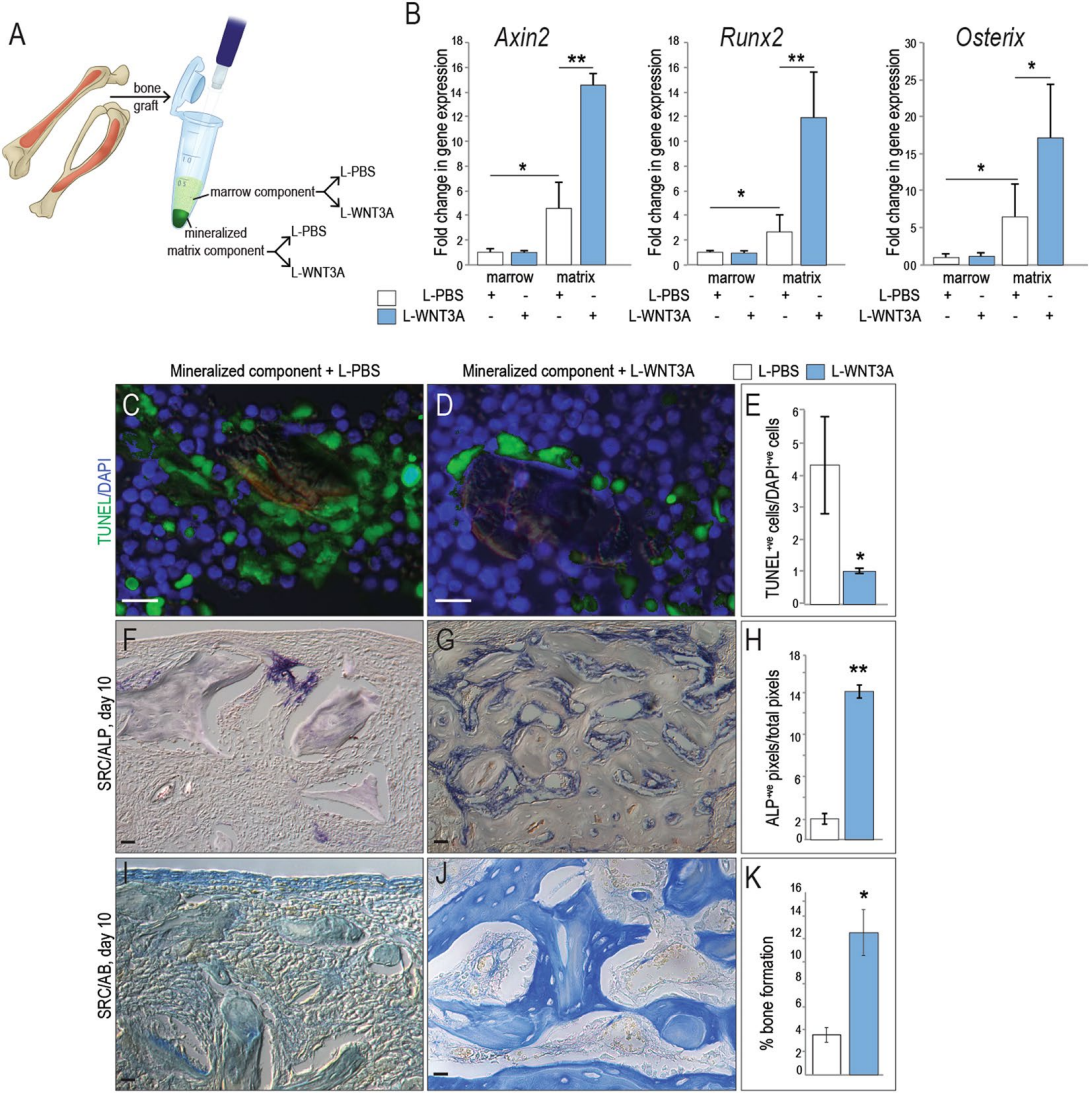
L-WNT3A treated autografts exhibit enhanced survival and osteogenesis. (**A**) Experimental design, where bone grafts were harvested, separated into mineralized matrix and marrow components then treated with either L-PBS or L-WNT3A. (**B**) qRT-PCR analyses of both components, showing fold-change in expression of *Axin2*, *Runx2*, and *Osterix* 24 h after treatment with L-PBS (white bars) or L-WNT3A (grey bars). TUNEL staining on representative tissue sections derived from aliquots of the mineralized matrix component of a bone graft treated with either (**C**) L-PBS or (**D**) L-WNT3A for 1 h then transplanted to the SRC for 10 days. (**E**) Quantification of TUNEL^+ve^ cells/total number of viable, DAPI^+ve^ cells on representative tissue sections. ALP staining on representative tissue sections derived from aliquots of the mineralized matrix component of a bone graft treated with either (**F**) L-PBS or (**G**) L-WNT3A; (**H**) quantification of ALP^+ve^ pixels/total pixels. Aniline blue staining on representative tissue sections derived from aliquots of the mineralized matrix component of a bone graft treated with either (**I**) L-PBS or (**J**) L-WNT3A; (**K**) quantification of new bone volume. Scale bars = 50 µm. Asterisk indicates p-value < 0.05., double asterisks indicate p-value < 0.01.

Our data suggest that at least part of the reason why a bone graft loses its efficacy is related to a decline in endogenous Wnt signaling. We tested whether treatment of a bone graft with WNT3A protein could restore osteogenic capacity back to bone grafts from aged animals. To do this, bone grafts were harvested from mice, separated into marrow and mineralized matrix components, and exposed to liposome-reconstituted WNT3A (L-WNT3A^25,26^) or control, L-PBS (Fig. 4A). Quantitative RT-PCR analyses demonstrated that in response to L-WNT3A, the marrow component showed no detectable change in expression of *Axin2* (Fig. 4B). On the other hand, cells in the mineralized matrix component responded robustly to the L-WNT3A stimulus by up regulating the Wnt target gene *Axin2* (Fig. 4B).

The L-WNT3A stimulus was sufficient to up regulate osteogenic gene expression. Compared to the marrow fraction, baseline expression of *Runx2* and *Osterix* was significantly higher in the mineralized matrix fraction (compare white bars, Fig. 4B). In response to the L-WNT3A stimulus, cells in the mineralized matrix component responded by up regulating *Runx2* and *Osterix* nearly 10-fold over baseline (Fig. 4B).

The biological effect of L-WNT3A treatment was further interrogated, by introducing the mineralized matrix component of the bone graft into the SRC. Compared with L-PBS, apoptosis in the mineralized component was curtailed by L-WNT3A treatment (Fig. 4C,D; quantified in E). Over time, L-WNT3A treatment resulted in significantly increased ALP activity (Fig. 4F,G; quantified in H), and significantly increased osteoid matrix production in the mineralized matrix component of a bone graft (Fig. 4I,J; quantified in K).

## Discussion

The initial goal of this work was to understand what features make an autologous bone graft effective at generating new bone; our analyses conclusively point to the mineralized component of an autograft as being critically important for its osteogenic capacity (Fig. 1). The data on the mineralized matrix of a bone graft are particularly relevant when considering the work of Bruchardt and colleagues, who thirty years ago speculated on the origins of the new bone arising from an autograft^27^. Bruchardt favored an interpretation where the cells responsible for depositing new bone matrix (a process referred to then as “creeping substitution”) were associated with the dead bone matrix^27^. Using lineage tracing methods and the SRC assay, we now verify that in an autograft, the new bone arises from the differentiation of Wnt responsive cells associated with the dead bone matrix.

The mineralized component of a bone graft diminishes with age. For example, aging is characterized by a loss in cancellous (trabecular) bone volume^28^, which is visualized as an overall decline in trabecular number and thickness^29^. We observe the same loss of trabecular architecture in aging animals (Fig. 3). Since most of the bone chips in an autograft arise from cancellous bone^30^, aging has an indirect, yet nevertheless important impact on the mineralized component of an autograft.

Endogenous Wnt signaling declines with age. Osteopenia and osteoporosis are both characterized by a pathologic reduction in bone mineral density and its etiology is strongly associated with a reduction in endogenous Wnt signaling^31^. Reduced endogenous Wnt signaling is also seen in loss-of-function mutations in the human Wnt receptor LRP5, which cause low bone mass diseases^32^. In human bone samples, high-throughput RNA sequencing analyses demonstrate that the Wnt target gene *Lef1* is expressed at significantly lower levels in bones from aged versus young women^33^. The premature aging syndrome, progeria, is associated with an inhibition of Wnt signaling caused by reduced nuclear localization and impaired transcriptional activity of *Lef1* ^34^. The fact that progeria mice exhibit accelerated bone mineral density loss^34^ lends strong support for a relationship between aging, reduced Wnt signaling, and diminished bone-forming potential.

Clinical trials are now underway testing the efficacy of an anti-Sclerostin antibody for osteoporosis^35^. Sclerostin is a negative regulator of Wnt signaling^36^ and by inhibiting its action, the result in an up regulation of Wnt signaling. Early data indicate that patients show a significant increase in bone accrual^35^, lending further support for a means to encourage bone formation by increasing Wnt signaling. Side effects however, have become obvious: clinical trial data demonstrate that inhibiting Sclerostin leads to a compensatory increase in DKK-1 expression, which may limit the anabolic effects of Sclerostin blockade on bone formation^37^. Moreover, some patients developed neutralizing antibodies during the course of anti-Sclerostin antibody treatment^38^. Recent data released from the ARCH study also show that treatment with the anti-Sclerostin antibody romosozumab is associated with more cardiovascular events than with alendronate^39^. Clearly, systemic methods to amplify Wnt signaling must prove to be safe before they can be widely employed.

Anti-Sclerostin and anti-DKK1 strategies use an “inhibit an inhibitor” approach to elevate Wnt signaling and at best can only return endogenous Wnt signaling to baseline levels. Since endogenous Wnt signaling declines with age (Fig. 3 and see ref.^40^), returning Wnt signaling to baseline may be insufficient when it comes to stimulate bone production^33^.

Our strategy was therefore to transiently amplify Wnt signaling, specifically in a bone graft. Most Wnt responsive cells in an autograft are associated with the mineralized matrix component (Fig. 2), and endogenous Wnt signaling declines with age (Fig. 3; see also refs^7,10,31,41,42^). Collectively, these data suggest that expanding the Wnt responsive population in a bone graft from an aged animal may restore its bone-forming capacity.

Of the 19 Wnt ligands, Wnt3a protein exhibits one of the highest rates of secretion, making it one of the easiest Wnts to produce and purify^43^. Wnt3a is also naturally expressed in bone grafts, and its level of expression declines with age^7^. Wnt3a acts through a beta catenin-dependent pathway^44^, and based on ligand-receptor binding studies Wnt3a shows high affinity to the Frizzled/Lrp complex^45^. Therefore, we used the recombinant Wnt3a protein as a means to activate Wnt signaling in bone graft material.

We showed that treatment with L-WNT3A elevated endogenous Wnt signaling in the bone graft (Fig. 4B), leading to reduced apoptosis of cells in the autograft (Fig. 4C–E and see refs^7,10^). L-WNT3A also reduces apoptosis in a bone graft, which directly impacts the number of engrafting cells at the site of transplantation^25^. L-WNT3A also transiently stimulates proliferation of cells in the bone graft^10^ but the pro-mitotic effect is temporary; within 7 days of a single treatment, cell proliferation returns to baseline levels^10^. Also, it should be noted that only cells in the autograft- and not the surrounding tissues- become activated by L-WNT3A, which confers a measure of safety to the use of a potent stem cell activator.

An *ex vivo* therapeutic strategy for amplifying Wnt signaling has a built-in safety margin. Rather than introducing a potent stem cell activator directly into the human body - which for recombinant human bone morphogenetic protein 2 (BMP2) has led to life-threatening complications^46^- we tested a method wherein a patient’s autograft is harvested as part of a standard operating room procedure then, while the surgeon prepares the recipient site, the autograft is combined with L-WNT3A. The resulting bone graft give rises to three times more bone than untreated samples (Fig. 4I–K). Thus, L-WNT3A represents an innovative biologic for bone tissue engineering, specifically geared towards an at-risk population of elderly patients.

## Electronic supplementary material

Supplemental Figure
