# Supplementary material for: A WNT protein therapeutic improves the bone-forming capacity of autografts from aged animals
Source: Sci Rep. 2018 Jan 8;8:119. doi: 10.1038/s41598-017-18375-x (PMC5758817; doi:10.1038/s41598-017-18375-x)
Supplement: Supplementary file 1 — Supplemental Figure [file 41598_2017_18375_MOESM1_ESM.pdf]

A WNT protein therapeutic improves the bone-forming capacity of autografts from aged animals

Tao Chen[1,2,*], D.D.S., Ph.D.; Jingtao Li[2,3,*], D.D.S., Ph.D.; Luis A. Córdova, DDS, PhD.[2,4]; Bo Liu[2,5], D.D.S., Ph.D.; Sylvain Mouraret[2,6], DDS; Qiang Sun, MD[2,7]; Benjamin Salmon[2,8], D.D.S., Ph.D.; and Jill Helms[2,5, #], D.D.S., Ph.D.

1 Stomatological Hospital of Chongqing Medical University, Chongqing Key Laboratory of Oral Diseases and Biomedical Sciences, Chongqing Municipal Key Laboratory of Oral Biomedical Engineering of Higher Education, Chongqing, 400000, China

2 Division of Plastic and Reconstructive Surgery, Department of Surgery, Stanford School of Medicine, Stanford, CA 94305

3 State Key Laboratory of Oral Diseases & National Clinical Research Center for Oral Disease & Department of Oral Maxillofacial Surgery, West China Hospital of Stomatology, Sichuan University, Chengdu, 610007, China

4 Department of Oral and Maxillofacial Surgery, Faculty of Dentistry, University of Chile, Santiago, Chile

5 Ankasa Regenerative Therapeutics, Inc. 329 Oyster Point Blvd. Suite 3306, South San Francisco, CA 94080

6 Department of Periodontology, Service of Odontology, Rothschild Hospital, AP-HP, Paris

7 – Denis, Diderot University, U.F.R. of Odontology, Paris, France

7 Department of Plastic Surgery, The First Hospital of China Medical University, Shenyang, 110001 China

8 Paris Descartes - Sorbonne Paris Cite University, Dental School, EA2496, Montrouge, France and Dental Medicine Department, Bretonneau Hospital, HUPNVS, AP-HP, Paris, France.

*Authors contributed equally.

# Corresponding author:

Jill A. Helms, Stanford University, 257 Campus Dr., Stanford, CA 94305, USA.

Email: jhelms@stanford.edu

One supplemental figure has been included.

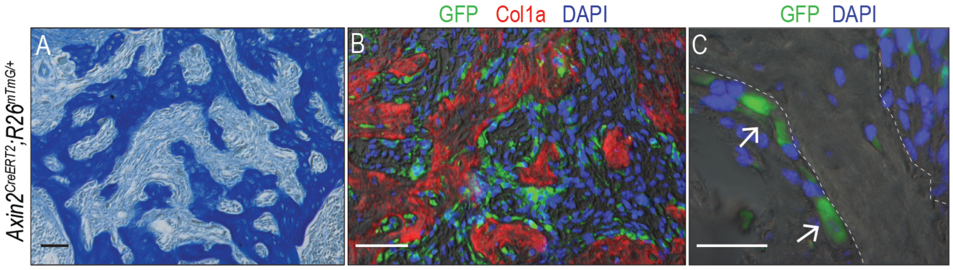

Supplemental Figure 1. Wnt responsive cells contribute to the bone repair.

(A) Representative tissue sections through a tibial defect produced in *Axin2*$^{CreERT2/+}$;*R26*$^{mTmG/+}$ mice, where (A) Aniline blue staining on post-injury day 7 identifies new bone in the injury site. On an adjacent tissue section (B) GFP immunostaining identifies Wnt responsive cells lining the new bone matrix that co-immunostained for Collagen Type I. (C) Higher magnification showing GFP^{+ve} cells on the surface of new bone. Scale bars = 50μm.

Supplemental Table 1. Experimental groups

Test group	N	Control group	N	Genotype/ species	Analyses	Shown in:
Mineralized matrix component	3 for Histology and IHC; 3 for qRT-PCR; 3 for SRC	Marrow component	3 for Histology and IHC; 3 for qRT-PCR; 3 for SRC	n/a	Histology, IHC, qRT-PCR, and SRC transplantation	Fig. 1
Marrow component + DBM	3	Marrow component	3	Beta actin-GFP donor, syngeneic host	Histology, DAPI, ALP, GFP immunostaining	Fig. 1
Mineralized matrix component	3 for intact; 3 for SRC	Marrow component	3 for intact; 3 for SRC	*Axin2*$^{LacZ/+}$	Xgal	Fig. 2
Mineralized matrix component	3 for Flow cytometry; 3 for IHC, GFP immunostaining	Marrow component	3 for Flow cytometry; 3 for IHC, GFP immunostaining	*Axin2*$^{CreERT2/+}$; *R26*^{mTmG}	Flow cytometry, IHC, GFP immunostaining	Fig. 2
Tibial mono-cortical defect	3	Intact tibia	*3*	*Axin2*$^{LacZ/+}$ *and* *Axin2*$^{CreERT2/+}$; *R26*^{mTmG}	Histology, GFP immunostaining	Fig. 2
Marrow component	3 for IHC and histology; 3 for	Marrow component	3 for IHC and histology; 3 for	Wild-type, *Axin2*$^{LacZ/+}$	IHC, histology, qRT-PCR, μCT, Xgal	Fig. 3

, aged donor	qRT-PCR; 3 for uCT; 3 for Xgal	young donor	qRT-PCR; 3 for uCT; 3 for Xgal			
Bone graft material, aged donor + L-WNT3A	4	Bone graft material, aged donor + L-PBS	*4*	Wild-type	qRT-PCR	Fig. 4
Mineralized matrix, aged donor + L-WNT3A	4	Mineralized matrix, aged donor + L-PBS	*4*	Wild-type	histology, ALP, TUNEL	Fig. 4
